# Mendelian Randomization Analysis Reveals a Causal Effect of Urinary Sodium/Urinary Creatinine Ratio on Kidney Function in Europeans

**DOI:** 10.3389/fbioe.2020.00662

**Published:** 2020-07-07

**Authors:** Yue-miao Zhang, Jie Zheng, Tom R. Gaunt, Hong Zhang

**Affiliations:** ^1^Renal Division, Peking University First Hospital, Peking University Institute of Nephrology, Key Laboratory of Renal Disease, Ministry of Health of China, Key Laboratory of Chronic Kidney Disease Prevention and Treatment (Peking University), Ministry of Education, Beijing, China; ^2^MRC Integrative Epidemiology Unit (IEU), Bristol Medical School, University of Bristol, Oakfield House, Bristol, United Kingdom; ^3^National Institute for Health Research (NIHR), Bristol Biomedical Research Centre, Bristol, United Kingdom

**Keywords:** salt restriction, urinary sodium/urinary creatinine ratio, kidney function, Mendelian randomization, causal effect

## Abstract

Salt restriction was recommended in clinical practice guideline for chronic kidney disease (CKD) treatment, but its effect on kidney outcomes remains conflicting. We aimed to test the causal effect of salt intake, using estimated 24-h sodium excretion from spot urinary sodium/urinary creatinine (UNa/UCr) ratio as a surrogate, on renal function using two-sample Mendelian randomization (MR). Genetic instruments for UNa/UCr were derived from a recent genome-wide association study of 218,450 European-descent individuals in the UK Biobank. Kidney outcomes were creatinine-based estimated glomerular filtration rate (eGFRcrea) (*N* = 567,460) and CKD (eGFRcrea < 60 ml/min/1.73 m^2^, *N* cases = 41,395, *N* controls = 439,303) from the CKDGen consortium. Cystatin C-based eGFR (eGFRcys) and eGFRcrea single-nucleotide polymorphisms associated with blood urea nitrogen (BUN) were used for sensitivity analyses. MR revealed a causal effect of UNa/UCr on higher eGFRcrea [β = 0.14, unit change in log ml/min/1.73 m^2^ per UNa/UCr ratio; 95% confidence interval (CI) = 0.07 – 0.20, *P* = 2.15 × 10^–5^] and a protective effect against CKD risk (odds ratio = 0.24, 95% CI = 0.14 to 0.41, *P* = 1.20 × 10^–7^). The MR findings were confirmed by MR-Egger regression, weighted median MR, and mode estimate MR, with less evidence of existence of horizontal pleiotropy. Consistent positive causal effect of UNa/UCr on eGFRcys was also detected. On the other hand, bidirectional MR suggested inconclusive results of CKD, eGFRcrea, eGFRcrea (BUN associated), and eGFRcys on UNa/UCr. The average 24-h sodium excretion was estimated to be approximately 2.6 g per day for women and 3.7 g per day for men. This study provides evidence that sodium excretion, well above the recommendation of <2 g per day of sodium intake, might not have a harmful effect on kidney function. Clinical trials are warranted to evaluate the sodium restriction target on kidney function.

## Introduction

Chronic kidney disease (CKD) accounts for 10% to 15% population worldwide, which is associated with high annual rates of mortality and cardiovascular complications (Xie et al., 2018). Identifying potentially modifiable risk factors for CKD is critical in order to devise effective, population-based preventive strategies.

A salt restriction <90 mmol per day (equivalent to 5 g per day of salt or 2 g per day of sodium) is recommended in the 2012 Kidney Disease: Improving Global Outcomes (KDIGO) clinical practice guideline (Kidney Disease: Improving Global Outcomes [KDIGO] and CKD Work Group, 2012). The Working Group graded this recommendation as “1C,” indicating that controlling salt intake can be evaluated as a candidate for developing a policy or a performance measure (Grade level 1), but from low quality of evidence (Grade C). This recommendation was mainly based on expert opinion and extrapolation from previous studies between sodium intake and blood pressure. Observational epidemiology studies have indicated that sodium intake was positively associated with blood pressure and proteinuria, which are strong predictors of CKD progression and cardiovascular diseases (CVDs) (He et al., 2013). In addition, existing clinical trials in sodium restriction showed significant reductions in blood pressures and proteinuria (Vogt et al., 2008; Suckling et al., 2010; Slagman et al., 2011; Graudal et al., 2012; de Brito-Ashurst et al., 2013). However, prospective cohort studies have reported conflicting findings on the association between dietary sodium intake and CVD (Cook et al., 2007; O’Donnell et al., 2011, 2014). Moreover, emerging studies suggested that extreme strict sodium control may be harmful rather than beneficial as previously recommended in CKD patients (Fan et al., 2014). Debates about optimal sodium intake in the general population are still ongoing (Mente et al., 2016, 2018). During the 2017 KDIGO controversies conference, stakeholders questioned whether a level 1C recommendation of sodium restriction (<2 g of sodium per day) was too strong based on the current evidence (Cheung et al., 2019).

Given that few randomized controlled trials have been conducted to infer causality between salt intake and kidney outcomes, the genetic variants associated with potential risk factors offer the opportunity to test the causal effect of salt intake on CKD through Mendelian randomization (MR) (Smith and Ebrahim, 2003; Zheng et al., 2017). In essence, MR exploits the random allocation of genetic variants at conception and therefore is less susceptible to confounding compared to traditional observational studies. Furthermore, MR studies give the effects of lifetime exposures and so could evaluate the long-term effect. Recently, Zanetti et al. performed genome-wide association studies (GWASs) of urinary electrolyte excretion, including the urinary sodium/urinary creatinine (UNa/UCr) ratio, in up to 327,616 unrelated individuals of European ancestry from the UK Biobank (Zanetti et al., 2019). Since spot urine samples were commonly used to estimate 24-h sodium excretion as a surrogate for salt intake (Lee et al., 2013; Mente et al., 2016, 2018), we aimed to assess the causal effect of salt intake, using UNa/UCr as proxy, on kidney function in Europeans using MR (Hemani et al., 2018).

## Materials and Methods

### Genetic Predictors of Urinary Sodium Excretion

In a recent genetic study of urinary sodium excretion normalized for creatinine (calculated as UNa/UCr), 327,616 eligible individuals from the UK Biobank were included and two-thirds of the sample (*n* = 218,450) were randomly selected for discovery GWAS (Zanetti et al., 2019). Association analysis was conducted using linear regression of UNa/UCr levels on imputed genotypes, assuming an additive model between phenotype and genotype dosages. The model was adjusted for age, sex, batch, and the first 10 genotype principal components. Six conditional distinguished genetic variants strongly associated with UNa/UCr (*P* < 5 × 10^–8^) were selected as candidate instruments (Supplementary Table S1).

### Genetic Associations of Kidney Function Phenotypes

Creatinine-based estimated glomerular filtration rate (eGFRcrea) and CKD (eGFRcrea < 60 ml/min/1.73 m^2^, binary phenotype) were used as proxies for kidney function phenotypes. eGFRcrea was estimated from serum creatinine using the Chronic Kidney Disease Epidemiology Collaboration (CKD-EPI) formula (Levey et al., 2009). It was estimated as 141 × min(Scr/κ, 1)^α^ × max(Scr/κ, 1)^–1.209^ × 0.993^*Age*^ × 1.018 (if female)_1.159 (if black), where Scr is the serum creatinine, κ is 0.7 for females and 0.9 for males, α is -0.329 for females and -0.411 for males, min indicates the minimum of Scr/κ or 1, and max indicates the maximum of Scr/κ or 1. GWAS summary statistics of eGFRcrea (*N* = 567,460) and CKD (*N* cases = 41,395; *N* controls = 439,303; *N* total = 480,698) from the CKDGen consortium were utilized (Wuttke et al., 2019). The GWASs were imputed using the 1000 Genomes phase 1 v3 training set with 38 million variants. Genetic associations were adjusted for study specific covariates such as age and sex, and the first 10 principal components were also used as covariates in the association model of the GWAS to control for the population stratification.

### Mendelian Randomization

**FIGURE 1:**
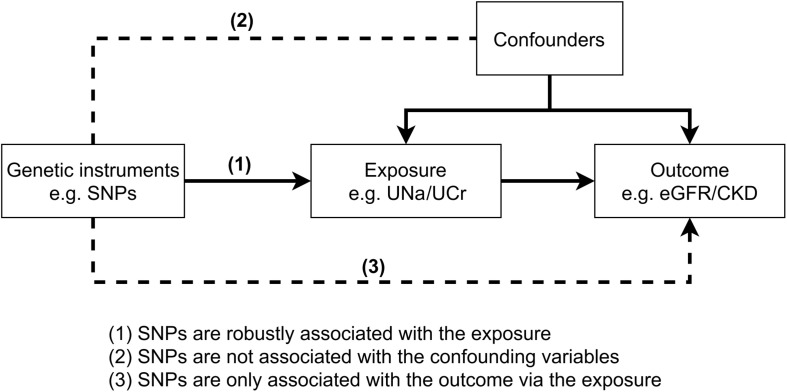
The diagram and three key assumptions of Mendelian randomization.

MR is an instrumental variable method that uses genetic variants as instruments to test the casual relationships between an exposure (e.g., UNa/UCr) and an outcome (e.g., eGFRcrea/CKD). The causal association between the exposure and the outcome will be valid when the three core MR assumptions were satisfied (Figure 1). In our initial MR analysis, UNa/UCr was treated as exposure and eGFRcrea/CKD as outcomes, using the UNa/UCr-associated single-nucleotide polymorphisms (SNPs) as instrument variables. We used the random-effect inverse variance weighted (IVW) method (Burgess et al., 2013) to estimate the MR effect, which pools the individual SNP Wald estimator (Lawlor et al., 2008) by meta-analysis. The Cochrane’s Q and Rocker’s Q statistics were used to estimate the level of heterogeneity of the IVW and MR-Egger regression separately, in which the degree of freedom for Cochrane’s Q is the number of SNPs minus one and that for Rocker’s Q is the number of SNPs minus two.

We further tested the MR assumptions using the following sensitivity analyses. MR-Egger regression (Bowden et al., 2015) was used to test pleiotropic effects of the instruments on outcomes, independent of exposures. The MR-Egger regression intercept was recorded as an indicator of pleiotropy. We also performed MR analyses using the weighted median MR approach (Bowden et al., 2016) and the mode estimate MR approach (Hartwig et al., 2017), which provides consistent causal estimates of the exposure on the outcome even when up to 50% (or even 0%) of the information contributing to the analysis comes from genetic variants that exhibit pleiotropy. If all approaches (i.e., IVW MR, MR-Egger regression, weighted median MR, and mode estimate MR) provide consistent causal estimates of UNa/UCr on eGFRcrea/CKD, the MR findings will be more robust. To exclude the influence of pleiotropy instruments on the MR estimates, we applied MR-PRESSO (Verbanck et al., 2018) as another sensitivity analysis, which attempts to reduce heterogeneity in the estimate of the causal effect by removing SNPs that contribute to the heterogeneity disproportionately more than expected.

### Directionality Analysis

#### Steiger Filtering

When applying MR, we assume that SNPs used as proxies of UNa/UCr exert their primary association on UNa/UCr and that any correlation with eGFRcrea or CKD is a consequence of a causal effect of UNa/UCr on eGFRcrea or CKD. We therefore performed Steiger filtering analyses (Hemani et al., 2018), implemented in the TwoSampleMR R package, to test the directionality of UNa/UCr instruments. The underline concept of Steiger filtering is a simple inequality. Given that phenotype A causes phenotype B, then we would expect that

$$\sum_{i=1}^{M}c\,o\,r\,{\left(g\,i,A\right)}^{2}>\sum_{i=1}^{M}c\,o\,r\,{\left(g\,i,B\right)}^{2}$$

since *cor*(*g_*i*_, B*)^2^ = *cor*(*A*, *B*)^2^^∗^ *cor*(*g*_*i*_, *A*)^2^, where “*cor*” denotes correlation, and the vector *g* contains a set of SNPs that influence phenotype A. For any instrument that had a *cor*(*g*_*i*_, *A*)^2^ < *cor*(*g*_*i*_, *B*)^2^, they showed evidence of primarily affecting eGFR rather than UNa/UCr. We thus removed these UNa/UCr instruments and performed the MR analyses using the remaining instruments.

#### Bidirectional MR

To investigate the potential reverse causality of eGFRcrea/CKD on UNa/UCr, we conducted a bidirectional MR. We first found 256 conditional independent variants associated with eGFRcrea and 23 independent variants associated with CKD (Supplementary Table S2) using the linkage disequilibrium clumping (*r*^2^ < 0.001) function implemented in the TwoSampleMR R package. We then used eGFRcrea or CKD as exposures and UNa/UCr as the outcome for the bidirectional MR. Considering that eGFRcrea has GFR and marker determinants, we further performed sensitivity analyses by using blood urea nitrogen (BUN) GWAS (Wuttke et al., 2019) to prioritize eGFRcrea-associated SNPs as exposures. More specifically, we required the eGFRcrea SNPs be associated with BUN in the opposite direction (since higher GFR would lead to lower BUN) and the eGFRcrea SNPs be associated with BUN with a Bonferroni-corrected significance. In addition, cystatin C, an alternative biomarker of kidney function, was measured in a subset of European ancestry participants from the CKDGen consortium (*N* = 32,861) (Li et al., 2017). Cystatin C-based eGFR (eGFRcys) could be estimated as 76.7 × serum cystatin C (Levey et al., 2011). To further exclude the possible bias caused by creatinine, we extracted five SNPs that robustly associated with eGFRcys (Li et al., 2017) and used them as instruments for a sensitivity MR analysis against UNa/UCr.

### MR and Visualization Software

The MR results were presented as scatter plots and forest plots using a code derived from the ggplot2 package in R^1^. All MR and sensitivity analyses were conducted using the TwoSampleMR R package^2^ (Hemani et al., 2018). MR-PRESSO test was conducted by using the MR-PRESSO R package^3^. The number of distributions was set to 10,000, and the threshold was set to 0.05.

### 24-h Urinary Sodium Estimation

The exact intake of sodium cannot be measured precisely but can be estimated from 24-h sodium excretion (Kawano et al., 2007). Thus, to estimate the sodium intake, we used the INTERSALT (International Cooperative Study on Salt, Other Factors, and Blood Pressure) equation (Brown et al., 2013), developed in a sample of 3,093 European-descent individuals aged 20–59 years, to estimate the 24-h urine sodium excretion based on the reported spot urine measurements. The equation was described as follows.

For men:

24-h sodium excretion (mg) = 23 × {25.46 + [0.46 × spot urine sodium (mmol/L)] − [2.75 × spot urine creatinine (mmol/L)] − [0.13 × spot urine potassium (mmol/L)] + [4.10 × body mass index (kg/m^2^)] + [0.26 × age (years)]}.

For women:

24-h sodium excretion (mg) = 23 × {5.07 + [0.34 × spot urine sodium (mmol/L)] − [2.16 × spot urine creatinine (mmol/L)] − [0.09 × spot urine potassium (mmol/L)] + [2.39 × body mass index (kg/m^2^)] + [2.35 × age (years)] − [0.03 × age^2^ (years)]}.

## Results

### Effect of UNa/UCr on eGFRcrea and CKD

**FIGURE 2:**
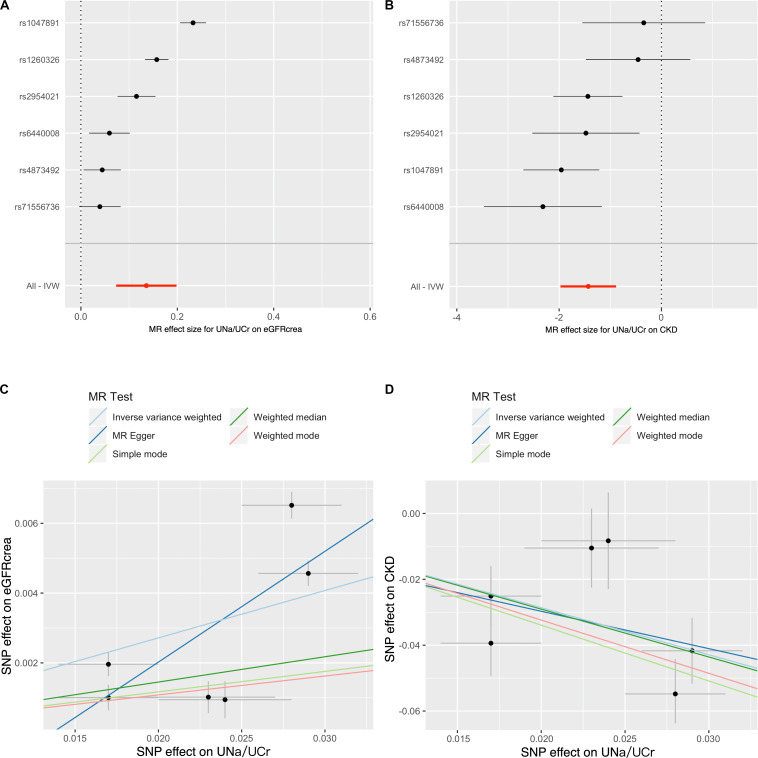
Forest plot and scatter plot for causal effects of urinary sodium/creatinine ratio on eGFRcrea and CKD. The forest plot shows the Wald ratio estimate of each UNa/UCr instrument on eGFRcrea **(A)** or CKD **(B)**. The scatter plots display the causal estimates of UNa/UCr (*x*-axis) against eGFRcrea **(C)** or CKD **(D)**. Each point refers to one UNa/UCr instrument. The slopes of the lines represent the causal estimates of UNa/UCr on CKD or eGFRcrea using inverse variance weighted, MR-Egger regression, weighted median, and mode estimate approaches. The *y*-intercept of the MR-Egger regression line is an estimate of the degree of horizontal pleiotropy in the dataset. Notation: CKD, chronic kidney disease; eGFRcrea, creatinine-based estimated glomerular filtration rate; IVW, inverse variance weighted; UNa/UCr, urinary sodium/urinary creatinine ratio.

Figure 2 showed the MR results of UNa/UCr on eGFRcrea and CKD. For eGFRcrea, the IVW MR suggested that UNa/UCr was positively associated with eGFRcrea [β = 0.14, unit change in log ml/min/1.73 m^2^ per UNa/UCr ratio; 95% confidence interval (CI) = 0.07 – 0.20, *P* = 2.15 × 10^–5^, Figure 2A]. Similarly, the IVW MR suggested that increased UNa/UCr was associated with reduced CKD risk [odds ratio (OR) = 0.24, 95% CI = 0.14 to 0.41, *P* = 1.20 × 10^–7^, Figure 2B]. The SNP MR results presented by the forest plots suggested that the causal effects of UNa/UCr on eGFRcrea/CKD were similar across instruments, and the observed MR findings were not massively influenced by any single instrument (Figures 2A,B). Figures 2C,D showed scatter plots of the SNP-outcome associations against the SNP-UNa/UCr associations, allowing visualization of the causal effect for individual SNP on eGFRcrea/CKD.

Cochrane’s Q test (*P* = 7.03 × 10^–7^ and 5.83 × 10^–3^ for eGFRcrea and CKD as outcomes, respectively) and Rocker’s *Q*-test (*P* = 6.72 × 10^–7^ and 2.29 × 10^–3^ for eGFRcrea and CKD as outcomes, respectively) suggested the presence of considerable heterogeneity in the analysis. However, the MR-Egger intercept suggested little evidence of horizontal pleiotropy (*P* = 0.65 and 0.98 for eGFRcrea and CKD as outcome, respectively) (Supplementary Table S3).

### Sensitivity Analyses

Table 1 showed the MR estimates of UNa/UCr on eGFRcrea and CKD using other sensitivity MR approaches, which are more robust to pleiotropy. For eGFRcrea, the IVW MR result was consistent with the casual estimates using MR-Egger regression (β = 0.32, 95% CI = 0.06 – 0.58, *P* = 1.66 × 10^–2^), weighted median MR (β = 0.07, 95% CI = 0.04 – 0.11, *P* = 8.68 × 10^–6^), and mode-based estimate MR approaches (β = 0.05, 95% CI = 0.02 – 0.09, *P* = 1.82 × 10^–2^), which increased the robustness of the MR results. For CKD, the IVW MR estimates were concordant in direction with the MR estimates using MR-Egger (OR = 0.32, 95% CI = 0.02 – 4.97, *P* = 0.46), weighted median MR (OR = 0.23, 95% CI = 0.14 – 0.40, *P* = 1.40 × 10^–7^), and mode-based MR approaches (OR = 0.20, 95% CI = 0.10 – 0.41, *P* = 7.55 × 10^–3^). In consistent with these results, we also observed positive causal effect of UNa/UCr on eGFRcys (Supplementary Table S4). Given that some of the instruments might be influenced by horizontal pleiotropy, we applied the MR-PRESSO outlier test and identified three UNa/UCr instruments as potential outliers. The MR estimate after removing the outliers identified by MR-PRESSO suggested a consistent positive effect of UNa/UCr on eGFRcrea (MR-PRESSO estimate: β = 0.129, 95% CI = 0.076 – 0.182, *P* = 4.14 × 10^–2^; Supplementary Table S5).

**TABLE 1:** Mendelian randomization analyses of causal effects of urinary sodium/urinary creatinine ratio on eGFRcrea and CKD.

**Trait**	**Number of SNPs**	**Methods**	**Estimator (95% CI)**	***P***
eGFRcrea	6	MR IVW	β = 0.14 (0.07 – 0.20)	2.15 × 10^–5^
	6	MR-egger	β = 0.32 (0.06 – 0.58)	1.66 × 10^–2^
	6	MR-weighted median	β = 0.07 (0.04 – 0.11)	8.68 × 10^–6^
	6	MR-weighted mode	β = 0.05 (0.02 – 0.09)	1.82 × 10^–2^
CKD	6	MR IVW	OR = 0.24 (0.14 – 0.41)	1.20 × 10^–7^
	6	MR-egger	OR = 0.32 (0.02 – 4.97)	0.46
	6	MR-weighted median	OR = 0.23 (0.14 – 0.40)	1.40 × 10^–7^
	6	MR-weighted mode	OR = 0.20 (0.10 – 0.41)	7.55 × 10^–3^

*The confidence interval of MR-Egger estimates was wider compared to that of other methods because the statistical power of the MR-Egger was, in general, lower than that of MR IVW and weighted median. CKD, chronic kidney disease; eGFRcrea, creatinine-based estimated glomerular filtration rate; MR, Mendelian randomization; IVW, inverse variance weighted, OR, odds ratio; SNP, single-nucleotide polymorphism.*

### Directionality Tests

**FIGURE 3:**
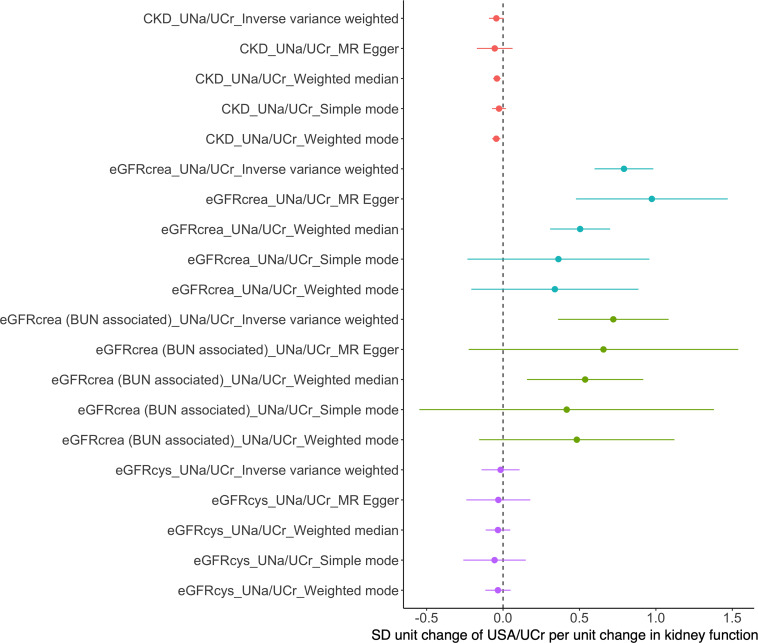
Mendelian randomization analyses of causal effects of eGFR/CKD on urinary sodium/creatinine ratio. BUN, blood urea nitrogen; eGFRcrea, creatinine-based estimated glomerular filtration rate; eGFRcrea (BUN associated), the eGFRcrea-associated SNPs were required to be associated with BUN in opposite direction (since higher GFR would lead to lower BUN) and to be associated with BUN with a Bonferroni-corrected significance; eGFRcys, cystatin C-based estimated glomerular filtration rate.

The Steiger filtering results suggested that all instruments were primarily influencing UNa/UCr than affecting eGFRcrea as a consequence (Supplementary Table S6), which further confirmed the robustness of the instruments. As shown in Figure 3, bidirectional MR using CKD, eGFRcrea, eGFRcrea (BUN associated), and eGFRcys as exposures and UNa/UCr as an outcome suggested inconsistent evidence of causal effect of kidney function on UNa/UCr (Supplementary Table S7).

### Sodium Intake Estimation

The mean age, body mass index, UNa/UCr, and urinary potassium/UCr were 56.88, 27.39, 10.60, and 8.48, respectively, in the discovery population and were 56.87, 27.42, 10.62, and 8.44, respectively, in the replication population (Zanetti et al., 2019). Using these baseline characteristics, the average 24-h sodium excretion was estimated to be approximately 2.6 g per day for women and 3.7 g per day for men, well above the recommended restriction of <2 g per day of sodium intake (Supplementary Table S8).

## Discussion

Sodium intake was reported to be one of the most important modifiable risk factors influencing blood pressure and proteinuria, yet its impact on renal outcomes is uncertain. Using a two-sample MR approach, we tested the causal effect of sodium intake, using estimated 24-h sodium excretion from a spot UNa/UCr ratio as a surrogate, on kidney function. We found that each unit increase in the UNa/UCr ratio was associated with a 0.14 unit increase in log(eGFRcrea) and with reduced CKD risk (OR = 0.24). These MR findings were confirmed by MR-Egger regression, weighted median MR, and mode estimate MR, which suggested less evidence of existence of horizontal pleiotropy. Steiger filtering suggested less evidence of reverse causality of CKD on UNa/UCr. Cochrane’s *Q* and Rocker’s *Q* statistics suggested the presence of considerable heterogeneity in the analysis. However, the MR-Egger intercept suggested little evidence of horizontal pleiotropy. Consistent positive causal effect of UNa/UCr on eGFRcys was also detected. Bidirectional MR using CKD, eGFRcrea, eGFRcrea (BUN associated), and eGFRcys as exposures showed weak evidence supporting a causal relationship for kidney function on UNa/UCr. The average 24-h sodium excretion was estimated to be approximately 2.6 g per day for women and 3.7 g per day for men, well above the recommendation of <2 g per day of sodium intake in the KDIGO guideline. Our results support that reducing dietary sodium intake under 2 g per day in CKD might be overcontrolled and may not have a beneficial effect on reducing CKD risk as expected.

Sodium intake was regarded as one of the important modifiable risk factors for CKD progression. The exact intake of sodium cannot be measured precisely but can be estimated from dietary questionnaires or 24-h sodium excretion (Kawano et al., 2007). However, questionnaires are usually self-reported and require calculations by a nutritionist and thus may be imperfect measures of sodium intake (Leiba et al., 2005), while the 24-h urine sample collection is limited to poor compliance and cost. Thus, studies usually used spot urine samples to estimate 24-h sodium excretion as a surrogate for sodium intake (Mente et al., 2016, 2018). To compensate for variation in urine concentration, urinary sodium corrected by urinary creatinine could be used for assessing sodium excretion in urine over 24-h (Lee et al., 2013). Using these methods, previous observational studies have provided inconsistent results on the effects of dietary sodium intake on the risk of CKD. Some studies suggested that urinary sodium excretion was associated with increased risk of CKD progression and incidence of end-stage renal disease (ESRD) (Mc Causland et al., 2012; Vegter et al., 2012; Ohta et al., 2013; Mc Quarrie et al., 2014), while others indicated that urinary sodium excretion was not associated with the risk of CKD or renal failure (Dunkler et al., 2013; Fan et al., 2014; Smyth et al., 2014), even protected against cumulative incidence of ESRD (Ekinci et al., 2011; Thomas et al., 2011). The inconsistent results may be due to the fact that these studies were observational studies that cannot make causal inference.

Until now, previous trials mainly focused on the blood pressure lowering effect of sodium control, but there are limited evidence detailing the effects of sodium restriction in CKD patients. For example, the Holland Nephrology Study (HON-EST) suggested that the addition of a low-sodium diet further reduced 11 mmHg systolic blood pressure in patients with treatment with an angiotensin converting enzyme inhibitor (Slagman et al., 2011). Vogt found reductions of 6/3 mmHg systolic blood pressure/diastolic blood pressure in proteinuric patients without diabetes who received a low-sodium diet (Vogt et al., 2008). Two more recent studies provided new clinical trial data from randomized, double-blind, crossover trials in patients with stages 3 and 4 CKD (McMahon et al., 2013; Saran et al., 2017). They found that salt restriction resulted in statistically significant reductions of blood pressure and albuminuria. However, although salt intake has been extensively suggested to be associated with blood pressure, high sodium intake-related increase in blood pressure might not entirely explain the elevated risk of CKD progression and renal failure in patients with CKD. What’s more, it must be noted that it is unable to compare the clinical outcomes among patients with urinary sodium excretion <2 g per day recommended in clinical practice guideline, which appears difficult to achieve. Results from most trials showed that only high urinary sodium excretion (approximately 150–200 mmol or 3.5–4.5 g sodium per day), well above the recommended limit (2 g sodium per day), was associated with increased risk of CKD progression or all-cause mortality (Norris et al., 2006; Lambers et al., 2012; McMahon et al., 2013; He et al., 2016). Thus, using these data is hard to assess whether sodium restriction <2 g per day is suggestively hazardous or intuitively advantageous (Anderson and Ix, 2013).

In the current study, we used the two-sample MR approach to test the causal effect of UNa/UCr on kidney outcomes. We further estimated the 24-h urine sodium excretion to estimate sodium intake using the previously published sodium excretion estimating equation based on spot urine measurements, which was developed and validated in European populations (Brown et al., 2013). We found that UNa/UCr showed causal effect on higher eGFRcrea and lower CKD risk, while weak evidence of causal association between kidney function and UNa/UCr was observed. Using baseline characteristics of the UK Biobank participants, the average 24-h sodium excretion was estimated to be approximately 2.6 g per day for women and 3.7 g per day for men. Previous studies suggested that this estimation equation might underestimate the urinary sodium excretion (Allen et al., 2017). Although potential bias exists in estimating 24-h sodium excretion using casual urinary test (Dougher et al., 2016), our results support that reducing dietary sodium intake under 2 g per day in CKD was too restrictive and might not be beneficial to reduce the risk of CKD development or progression as expected.

Several limitations should be mentioned in this study. First, 24-h urine collection was estimated rather than performed in the study population; target levels of UNa/UCr for protecting effects were not evaluated. In the future, additional large randomized controlled trials are required to determine the optimal target sodium intake. Second, we currently have limited access to the individual level genotype and phenotype data to perform further stratification of groups by instruments or outcomes and distributions. This type of analysis is needed to determine the optimal target of sodium intake in CKD in the future. Third, this study is limited to the only six instruments of UNa/UCr, even though they were derived from the well-powered and most recent UNa/UCr GWAS. Most of the current MR sensitivity analysis methods such as MR-Egger regression and weighted median need a large number of independent instrumental SNPs in order to test for pleiotropy. Thus, the MR results presented in this paper should be taken with caution. Fourth, the MR Steiger filtering approach has limitations, i.e., some levels of horizontal pleiotropy, where the SNP influences the outcome through some pathways other than the exposure, could induce problems because this is a means by which the instrument is invalid. Besides, some levels of unmeasured confounding between the exposure and the outcome could lead to inference of the wrong causal direction. We therefore applied a well-validated method MR-PRESSO. After controlling for pleiotropy instruments, our finding was still consistent. Finally, as in most epidemiological studies, MR assumes a linear relation between UNa/UCr and eGFR/CKD, which might not invariably be the case.

In conclusion, we found that a higher UNa/UCr ratio, which reflects proportionally higher salt intake, showed causal effects on higher eGFR and lower CKD risk. Further long-term studies to determine optimal target of sodium intake in CKD are required.

## Data Availability Statement

The kidney function datasets can be found from http://ckdgen.imbi.uni-freiburg.de/. The summary statistics of UNa/UCr was available at GRASP resource (https://grasp.nhlbi.nih.gov/FullResults.aspx, PMID: 30910378).

## Author Contributions

YZ, JZ, and HZ contributed to the conception and design of the study. YZ prepared the data and wrote the draft of the manuscript. JZ performed most of the statistical analysis. All authors contributed to manuscript revision and read and approved the submitted version.

## Conflict of Interest

The authors declare that the research was conducted in the absence of any commercial or financial relationships that could be construed as a potential conflict of interest.

## Footnotes

1 https://cran.r-project.org/web/packages/ggplot2/index.html

2 http://www.mrbase.org

3 https://github.com/rondolab/MR-PRESSO

## References

[B2] Allen N. B. Zhao L. Loria C. M. Van Horn L. Wang C. Y. Pfeiffer C. M. (2017). The validity of predictive equations to estimate 24-hour sodium excretion: the MESA and CARDIA urinary sodium study. *Am. J. Epidemiol.* 186 149–159. 10.1093/aje/kwx056 28838062 PMC5860382

[B3] Anderson C. A. Ix J. H. (2013). Sodium reduction in CKD: suggestively hazardous or intuitively advantageous? *J. Am. Soc. Nephrol.* 24 1931–1933. 10.1681/ASN.2013090923 24204000 PMC3839562

[B4] Bowden J. Davey S. G. Burgess S. (2015). Mendelian randomization with invalid instruments: effect estimation and bias detection through Egger regression. *Int. J. Epidemiol.* 44 512–525. 10.1093/ije/dyv080 26050253 PMC4469799

[B5] Bowden J. Davey S. G. Haycock P. C. Burgess S. (2016). Consistent estimation in Mendelian randomization with some invalid instruments using a weighted median estimator. *Genet. Epidemiol.* 40 304–314. 10.1002/gepi.21965 27061298 PMC4849733

[B6] Brown I. J. Dyer A. R. Chan Q. Cogswell M. E. Ueshima H. Stamler J. (2013). Estimating 24-hour urinary sodium excretion from casual urinary sodium concentrations in Western populations: the INTERSALT study. *Am. J. Epidemiol.* 177 1180–1192. 10.1093/aje/kwt066 23673246 PMC3664342

[B7] Burgess S. Butterworth A. Thompson S. G. (2013). Mendelian randomization analysis with multiple genetic variants using summarized data. *Genet. Epidemiol.* 37 658–665. 10.1002/gepi.21758 24114802 PMC4377079

[B8] Cheung A. K. Chang T. I. Cushman W. C. Furth S. L. Ix J. H. Pecoits-Filho R. (2019). Blood pressure in chronic kidney disease: conclusions from a Kidney Disease: improving global outcomes (KDIGO) controversies conference. *Kidney Int.* 95 1027–1036. 10.1016/j.kint.2018.12.025 31010478

[B9] Cook N. R. Cutler J. A. Obarzanek E. Buring J. E. Rexrode K. M. Kumanyika S. K. (2007). Long term effects of dietary sodium reduction on cardiovascular disease outcomes: observational follow-up of the trials of hypertension prevention (TOHP). *BMJ* 334 885–888. 10.1136/bmj.39147.604896.55 17449506 PMC1857760

[B10] de Brito-Ashurst I. Perry L. Sanders T. A. Thomas J. E. Dobbie H. Varagunam M. (2013). The role of salt intake and salt sensitivity in the management of hypertension in South Asian people with chronic kidney disease: a randomised controlled trial. *Heart* 99 1256–1260. 10.1136/heartjnl-2013-303688 23766446 PMC3756453

[B11] Dougher C. E. Rifkin D. E. Anderson C. A. Smits G. Persky M. S. Block G. A. (2016). Spot urine sodium measurements do not accurately estimate dietary sodium intake in chronic kidney disease. *Am. J. Clin. Nutr.* 104 298–305. 10.3945/ajcn.115.127423 27357090 PMC4962156

[B12] Dunkler D. Dehghan M. Teo K. K. Heinze G. Gao P. Kohl M. (2013). Diet and kidney disease in high-risk individuals with type 2 diabetes mellitus. *JAMA Intern. Med.* 173 1682–1692. 10.1001/jamainternmed.2013.9051 23939297

[B13] Ekinci E. I. Clarke S. Thomas M. C. Moran J. L. Cheong K. MacIsaac R. J. (2011). Dietary salt intake and mortality in patients with type 2 diabetes. *Diabetes Care* 34 703–709. 10.2337/dc10-1723 21289228 PMC3041211

[B14] Fan L. Tighiouart H. Levey A. S. Beck G. J. Sarnak M. J. (2014). Urinary sodium excretion and kidney failure in nondiabetic chronic kidney disease. *Kidney Int.* 86 582–588. 10.1038/ki.2014.59 24646858 PMC4149837

[B15] Graudal N. A. Hubeck-Graudal T. Jürgens G. (2012). Effects of low-sodium diet vs. high-sodium diet on blood pressure, renin, aldosterone, catecholamines, cholesterol, and triglyceride (Cochrane Review). *Am. J. Hypertens.* 25 1–15. 10.1038/ajh.2011.210 22068710

[B16] Hartwig F. P. Davey Smith G. Bowden J. (2017). Robust inference in summary data Mendelian randomization via the zero-modal pleiotropy assumption. *Int. J. Epidemiol.* 46 1985–1998. 10.1093/ije/dyx102 29040600 PMC5837715

[B17] He F. J. Li J. Macgregor G. A. (2013). Effect of longer term modest salt reduction on blood pressure: cochrane systematic review and meta-analysis of randomised trials. *BMJ* 346:f1325 10.1136/bmj.f1325 23558162

[B18] He J. Mills K. T. Appel L. J. Yang W. Chen J. Lee B. T. (2016). Urinary sodium and potassium excretion and CKD progression. *J. Am. Soc. Nephrol.* 27 1202–1212. 10.1681/ASN.2015010022 26382905 PMC4814179

[B19] Hemani G. Zheng J. Elsworth B. Wade K. H. Haberland V. Baird D. (2018). The MR-Base platform supports systematic causal inference across the human phenome. *eLife* 7:e34408 10.7554/eLife.34408 PMC5976434 29846171

[B20] Kawano Y. Tsuchihashi T. Matsuura H. Ando K. Fujita T. Ueshima H. (2007). Report of the working group for dietary salt reduction of the japanese society of hypertension: (2) Assessment of salt intake in the management of hypertension. *Hypertens. Res.* 30 887–893. 10.1291/hypres.30.887 18049019

[B21] Kidney Disease: Improving Global Outcomes (KDIGO) and CKD Work Group (2012). KDIGO 2012 clinical practice guideline for the evaluation, and management of chronic kidney disease. *Kidney Int. Suppl.* 3 1–150.

[B22] Lambers H. H. J. Holtkamp F. A. Parving H. H. Navis G. J. Lewis J. B. Ritz E. (2012). Moderation of dietary sodium potentiates the renal and cardiovascular protective effects of angiotensin receptor blockers. *Kidney Int.* 82 330–337. 10.1038/ki.2012.74 22437412

[B23] Lawlor D. A. Harbord R. M. Sterne J. A. C. Timpson N. Davey S. G. (2008). Mendelian randomization: using genes as instruments for making causal inferences in epidemiology. *Stat. Med.* 27 1133–1163. 10.1002/sim.3034 17886233

[B24] Lee S. G. Lee W. Kwon O. H. Kim J. H. (2013). Association of urinary sodium/creatinine ratio and urinary sodium/specific gravity unit ratio with blood pressure and hypertension: KNHANES 2009-2010. *Clin. Chim. Acta* 424 168–173. 10.1016/j.cca.2013.05.027 23751483

[B25] Leiba A. Vald A. Peleg E. Shamiss A. Grossman E. (2005). Does dietary recall adequately assess sodium, potassium, and calcium intake in hypertensive patients? *Nutrition* 21 462–466. 10.1016/j.nut.2004.08.021 15811766

[B26] Levey A. S. de Jong P. E. Coresh J. El Nahas M. Astor B. C. Matsushita K. (2011). The definition, classification, and prognosis of chronic kidney disease: a KDIGO controversies conference report. *Kidney Int.* 80 17–28. 10.1038/ki.2010.483 21150873

[B27] Levey A. S. Stevens L. A. Schmid C. H. Zhang Y. L. Castro A. F. III Feldman H. I. (2009). A new equation to estimate glomerular filtration rate. *Ann. Intern. Med.* 150 604–612. 10.7326/0003-4819-150-9-200905050-00006 19414839 PMC2763564

[B28] Li M. Li Y. Weeks O. Mijatovic V. Teumer A. Huffman J. E. (2017). SOS2 and ACP1 loci identified through large-scale exome chip analysis regulate kidney development and function. *J. Am. Soc. Nephrol.* 28 981–994. 10.1681/ASN.2016020131 27920155 PMC5328154

[B29] Mc Causland F. R. Waikar S. S. Brunelli S. M. (2012). Increased dietary sodium is independently associated with greater mortality among prevalent hemodialysis patients. *Kidney Int.* 82 204–211. 10.1038/ki.2012.42 22418981 PMC3779624

[B30] Mc Quarrie E. P. Traynor J. P. Taylor A. H. Freel E. M. Fox J. G. Jardine A. G. (2014). Association between urinary sodium, creatinine, albumin, and long-term survival in chronic kidney disease. *Hypertension* 64 111–117. 10.1161/HYPERTENSIONAHA.113.03093 24732890

[B31] McMahon E. J. Bauer J. D. Hawley C. M. Isbel N. M. Stowasser M. Johnson D. W. (2013). A randomized trial of dietary sodium restriction in CKD. *J. Am. Soc. Nephrol.* 24 2096–2103. 10.1681/ASN.2013030285 24204003 PMC3839553

[B32] Mente A. O’Donnell M. Rangarajan S. Dagenais G. Lear S. McQueen M. (2016). Associations of urinary sodium excretion with cardiovascular events in individuals with and without hypertension: a pooled analysis of data from four studies. *Lancet* 388 465–475. 10.1016/S0140-6736(16)30467-6 27216139

[B33] Mente A. O’Donnell M. Rangarajan S. McQueen M. Dagenais G. Wielgosz A. (2018). Urinary sodium excretion, blood pressure, cardiovascular disease, and mortality: a community-level prospective epidemiological cohort study. *Lancet* 392 496–506. 10.1016/S0140-6736(18)31376-X 30129465

[B34] Norris K. Bourgoigne J. Gassman J. Hebert L. Middleton J. Phillips R. A. (2006). Cardiovascular outcomes in the African American Study of Kidney Disease and Hypertension (AASK) Trial. *Am. J. Kidney Dis.* 48 739–751. 10.1053/j.ajkd.2006.08.004 17059993

[B35] O’Donnell M. Mente A. Rangarajan S. McQueen M. J. Wang X. Liu L. (2014). Urinary sodium and potassium excretion, mortality, and cardiovascular events. *N. Engl. J. Med.* 371 612–623. 10.1056/NEJMoa1311889 25119607

[B36] O’Donnell M. J. Yusuf S. Mente A. Gao P. Mann J. F. Teo K. (2011). Urinary sodium and potassium excretion and risk of cardiovascular events. *JAMA* 306 2229–2238. 10.1001/jama.2011.1729 22110105

[B37] Ohta Y. Tsuchihashi T. Kiyohara K. Oniki H. (2013). High salt intake promotes a decline in renal function in hypertensive patients: a 10-year observational study. *Hypertens. Res.* 36 172–176. 10.1038/hr.2012.155 23051657

[B38] Saran R. Padilla R. L. Gillespie B. W. Heung M. Hummel S. L. Derebail V. K. (2017). A randomized crossover trial of dietary sodium restriction in stage 3-4 CKD. *Clin. J. Am. Soc. Nephrol.* 12 399–407. 10.2215/CJN.01120216 28209636 PMC5338699

[B39] Slagman M. C. Waanders F. Hemmelder M. H. Woittiez A. J. Janssen W. M. Lambers Heerspink H. J. (2011). Moderate dietary sodium restriction added to angiotensin converting enzyme inhibition compared with dual blockade in lowering proteinuria and blood pressure: randomised controlled trial. *BMJ* 343:d4366 10.1136/bmj.d4366 PMC3143706 21791491

[B40] Smith G. D. Ebrahim S. (2003). ‘Mendelian randomization’: can genetic epidemiology contribute to understanding environmental determinants of disease? *Int. J. Epidemiol.* 32 1–22. 10.1093/ije/dyg070 12689998

[B41] Smyth A. Dunkler D. Gao P. Teo K. K. Yusuf S. O’Donnell M. J. (2014). The relationship between estimated sodium and potassium excretion and subsequent renal outcomes. *Kidney Int.* 86 1205–1212. 10.1038/ki.2014.214 24918156

[B42] Suckling R. J. He F. J. Macgregor G. A. (2010). Altered dietary salt intake for preventing and treating diabetic kidney disease. *Cochrane. Database. Syst. Rev.* 8:CD006763 10.1002/14651858.CD006763.pub2 21154374

[B43] Thomas M. C. Moran J. Forsblom C. Harjutsalo V. Thorn L. Ahola A. (2011). The association between dietary sodium intake, ESRD, and all-cause mortality in patients with type 1 diabetes. *Diabetes Care* 34 861–866. 10.2337/dc10-1722 21307382 PMC3064042

[B44] Vegter S. Perna A. Postma M. J. Navis G. Remuzzi G. Ruggenenti P. (2012). Sodium intake, ACE inhibition, and progression to ESRD. *J. Am. Soc. Nephrol.* 23 165–173. 10.1681/ASN.2011040430 22135311 PMC3269916

[B45] Verbanck M. Chen C. Y. Neale B. Do R. (2018). Detection of widespread horizontal pleiotropy in causal relationships inferred from Mendelian randomization between complex traits and diseases. *Nat. Genet.* 50 693–698. 10.1038/s41588-018-0099-7 29686387 PMC6083837

[B46] Vogt L. Waanders F. Boomsma F. de Zeeuw D. Navis G. (2008). Effects of dietary sodium and hydrochlorothiazide on the antiproteinuric efficacy of losartan. *J. Am. Soc. Nephrol.* 19 999–1007. 10.1681/ASN.2007060693 18272844 PMC2386733

[B47] Wuttke M. Li Y. Li M. Sieber K. B. Feitosa M. F. Gorski M. (2019). A catalog of genetic loci associated with kidney function from analyses of a million individuals. *Nat. Genet.* 51 957–972. 10.1038/s41588-019-0407-x 31152163 PMC6698888

[B48] Xie Y. Bowe B. Mokdad A. H. Xian H. Yan Y. Li T. (2018). Analysis of the Global Burden of Disease study highlights the global, regional, and national trends of chronic kidney disease epidemiology from 1990 to 2016. *Kidney Int.* 94 567–581. 10.1016/j.kint.2018.04.011 30078514

[B49] Zanetti D. Rao A. Gustafsson S. Assimes T. L. Montgomery S. B. Ingelsson E. (2019). Identification of 22 novel loci associated with urinary biomarkers of albumin, sodium, and potassium excretion. *Kidney Int.* 95 1197–1208. 10.1016/j.kint.2018.12.017 30910378 PMC6535090

[B50] Zheng J. Baird D. Borges M. C. Bowden J. Hemani G. Haycock P. (2017). Recent developments in mendelian randomization studies. *Curr. Epidemiol. Rep.* 4 330–345. 10.1007/s40471-017-0128-6 29226067 PMC5711966

